# Evaluating Fertilizer-Drawn Forward Osmosis Performance in Treating Anaerobic Palm Oil Mill Effluent

**DOI:** 10.3390/membranes11080566

**Published:** 2021-07-28

**Authors:** Ruwaida Abdul Wahid, Wei Lun Ang, Abdul Wahab Mohammad, Daniel James Johnson, Nidal Hilal

**Affiliations:** 1 Department of Chemical and Process Engineering, Faculty of Engineering and Built Environment, Universiti Kebangsaan Malaysia, Bangi 43600, SGR, Malaysia; ruydaaw@gmail.com (R.A.W.); drawm@ukm.edu.my (A.W.M.); 2 Centre for Sustainable Process Technology (CESPRO), Faculty of Engineering and Built Environment, Universiti Kebangsaan Malaysia, Bangi 43600, SGR, Malaysia; 3 NYUAD Water Research Center, New York University Abu Dhabi, P.O. Box 129188, Abu Dhabi, United Arab Emirates; djj2026@nyu.edu (D.J.J.); nidal.hilal@nyu.edu (N.H.)

**Keywords:** forward osmosis, fertilizer-drawn forward osmosis, draw solution, commercial fertilizer, palm oil mill effluent

## Abstract

Fertilizer-drawn forward osmosis (FDFO) is a potential alternative to recover and reuse water and nutrients from agricultural wastewater, such as palm oil mill effluent that consists of 95% water and is rich in nutrients. This study investigated the potential of commercial fertilizers as draw solution (DS) in FDFO to treat anaerobic palm oil mill effluent (An-POME). The process parameters affecting FO were studied and optimized, which were then applied to fertilizer selection based on FO performance and fouling propensity. Six commonly used fertilizers were screened and assessed in terms of pure water flux (J_w_) and reverse salt flux (J_S_). Ammonium sulfate ((NH_4_)_2_SO_4_), mono-ammonium phosphate (MAP), and potassium chloride (KCl) were further evaluated with An-POME. MAP showed the best performance against An-POME, with a high average water flux, low flux decline, the highest performance ratio (PR), and highest water recovery of 5.9% for a 4-h operation. In a 24-h fouling run, the average flux decline and water recovered were 84% and 15%, respectively. Both hydraulic flushing and osmotic backwashing cleaning were able to effectively restore the water flux. The results demonstrated that FDFO using commercial fertilizers has the potential for the treatment of An-POME for water recovery. Nevertheless, further investigation is needed to address challenges such as J_S_ and the dilution factor of DS for direct use of fertigation.

## 1. Introduction

Although the agricultural sector plays a major role in human affairs, it only contributed 3.3% of the world’s gross domestic product (GDP) in 2019 [1]. Agricultural development is an important tool to end extreme poverty and for securing global food supply. It has been projected that by 2050 the global population will reach 9.7 billion people [2] and this will require an estimated 50% increase in agricultural production. Agriculture accounts for the use of 70% of the world’s freshwater use [3]; hence, increases in food production would also increase water usage. Water usage in agriculture generates wastewater, which if untreated contributes to water pollution. It is estimated that 80% of all untreated wastewater is discharged into the world’s waterways [3], and the discharge of inadequately treated wastewater has an adverse impact on the environment and human health. The degradation of water quality of surface and groundwater due to pollution gravely affects water availability. Organic pollution can severely impact fisheries, livelihoods, and food security. Improper wastewater management also has a direct impact on ecosystems and the services they provide [4]. Nutrients (most importantly nitrogen, phosphorus, and potassium) and agrochemicals released from intensive agriculture and animal waste can further accelerate the eutrophication of freshwater and coastal marine ecosystems, which can lead to potentially toxic algal blooms and declines in biodiversity [3]. Thus, proper treatment of agriculture wastewater could mitigate these issues while providing alternative water resources.

The oil palm industry is one of the agricultural sectors that is experiencing substantial growth to meet the increasing demand for palm oil products, leading to an increase in the amount of biomass residues generated by the oil palm industry [5]. Associated with this growth, the discharge of palm oil mill effluent (POME), the wastewater generated from the mills, has increased, with approximately 3.50 tonnes of POME produced per tonne of crude palm oil production [6]. POME is non-toxic, acidic, and rich in organic matter and poses a severe threat of polluting the environment if discharged to waterways without treatment [7,8,9]. In recent years, efforts focused on developing a more sustainable palm oil milling operation. Water reclamation and bioresource recovery from POME are an appealing option towards a zero-discharge treatment system due to the characteristics of POME, which consists of 95–96% water and has an abundance of nutrients. Here, membrane technology has an edge over other treatment technologies as it can tackle pollutant removal, water reclamation, and bioresource recovery from POME, either individually or collectively. Appropriate membrane-based processing could deliver water quality suitable for use as irrigation and drinking water.

Recently, more studies are focusing on the potential of the forward osmosis (FO) filtration process in treating water and wastewater for nutrient or resource recovery [9,10,11]. The FO process is driven by the osmotic pressure differential across the membrane, rather than hydraulic pressure differential (as in reverse osmosis), for transport of water through the membrane. Since FO occurs spontaneously without hydraulic pressure, the process records lower energy consumption and fouling propensity, higher fouling reversibility and recovery [10,12,13]. The FO process results in concentration of a feed stream and dilution of a highly concentrated stream (referred to as the draw solution (DS), generally a salt solution, although many types of solute have been experimented with). Concentrated effluent that is rich in nutrients could be reused while the diluted DS can be used for direct application (depends on the type of draw solution) or clean water can be recovered from the diluted draw solution for reuse. One of the challenges of the FO process is the need to regenerate the DS for recycle or further use, which adds additional energy requirements and cost to the whole process [14,15]. Fertilizers as DS have an advantage over other salts as the diluted DS can be further used directly in irrigation systems. Studies have shown that fertilizer-drawn forward osmosis (FDFO) is feasible for water recovery and diluting fertilizers for direct application in fertigation or in aqua/hydroponic systems [16,17]. Choosing the right DS is important for the efficiency of the FO process. A suitable DS should be highly soluble in water, possess high osmotic pressure, low cost, and have good FO performance in terms of high water flux and low reverse salt flux (RSF) [18].

Similar to other membrane processes, FO is also susceptible to fouling. However, the fouling severity of FO membrane has been found to be lower, and the fouling is more reversible compared to reverse osmosis (RO). Jang et al. [19] stated that fouling in FO membranes was mainly due to cake-enhanced osmotic pressure (CEOP) phenomena caused by RSF. CEOP is a condition where severe flux declines and osmotic pressure near the membrane surface rises as a result of the back diffusion of salt from the membrane surface to the bulk solution that is hindered by the cake layer formed. However, the flux could be recovered by employing physical cleaning as simple as hydraulic flushing (adequate flux recovery) and osmotic backwashing (good flux recovery) [16]. A combination of both cleaning methods has showed that full flux restoration is achievable without the use of any chemical cleaning [20]. Nonetheless, the fouling severity and cleaning efficiency are highly variable subject to the constituents of the feed water (wastewater), and thus independent study should be conducted to obtain a better understanding of the performance of FO in handling various types of water.

Considering POME is a source for the recovery of water and nutrients but at the same time contains organic particulates, the potential of FDFO in recovering the resources, as well as the fouling propensity and cleaning efficiency of the process are worth investigation. This study explores the potential application of FDFO for the treatment of POME. Factors influencing the FO process, suitable fertilizers as DS, FO performance, and fouling propensity will be investigated for nutrient and water recovery from the FDFO process. The design of experiment (DoE) was applied to study and to optimize the process parameters affecting FO. The optimized parameters were then applied for DS selection based on FO performance, and the work was concluded with a fouling propensity study.

## 2. Materials and Methods

### 2.1. Membrane

A commercial flat-sheet cellulose triacetate (CTA) membrane, OsmoF_2_O^TM^, was acquired from Fluid Technology Solutions, Incorporated (FTSH_2_O, Albany, OR, USA) with embedded woven mesh structure. The intrinsic characteristics of the FO membrane shown in Table 1 (the water permeability coefficient (A), salt permeability coefficient (B) of the active layer, and structure parameter (S) of the support layer) were determined by adopting a protocol of a single FO experiment and were calculated using an Excel spreadsheet developed by Tiraferri et al. [21].

### 2.2. Feed and Draw Solutions

Deionized water (DI) was used as the feed solution (FS) for all basic FO performance experiments. All solutions used for the basic FO performance experiments were of reagent grade. For the FO performance experiment, sodium chloride (NaCl) was used as the draw solution (DS) for the baseline, and six fertilizers were used as DS (i.e., ammonium sulfate ((NH_4_)_2_SO_4_), ammonium nitrate (NH_4_NO_3_), monoammonium phosphate (MAP), diammonium phosphate (DAP), potassium chloride (KCl), and potassium nitrate (KNO_3_)). As for the FDFO performance experiment, anaerobically treated POME (An-POME) was used as feed, and three reagent-grade fertilizers (i.e., (NH_4_)_2_SO_4_, MAP and KCl) and three commercial grade chemical fertilizers (i.e., (NH_4_)_2_SO_4_-f, MAP-f and muriate of potash (KCl-f) were used as the DS. Draw solutions were prepared by dissolving fertilizer compounds in DI water. All chemicals were used directly without any further treatment except for KCl-f. KCl-f solution was centrifuged at 5000 rpm for 10 min [15] and then filtered through filter paper to remove undissolved particles/precipitants. The characteristics of the An-POME and details of the chemicals used are summarized in Table 2 and Table 3.

### 2.3. Lab-Scale FO System

**Figure 1:**
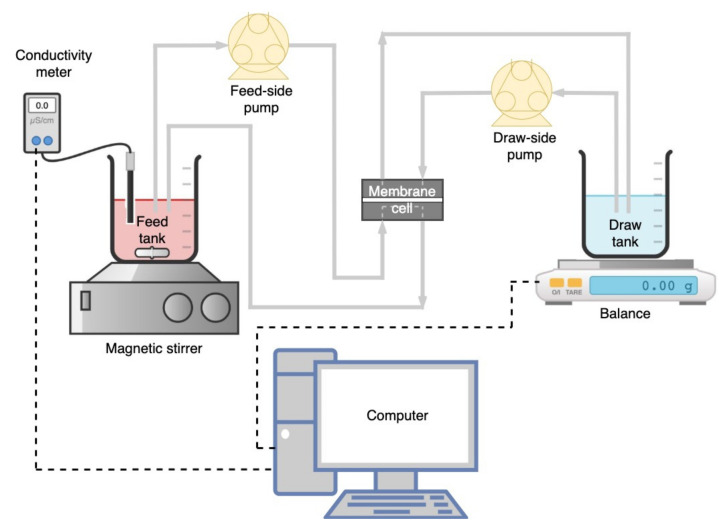
Schematic diagram of the forward osmosis (FO) filtration system.

A lab-scale FO filtration setup (Figure 1) consisting of a crossflow membrane module (Sterlitech CF042 Cell, Kent, WA, USA) with an effective membrane area of 0.0042 m^2^ was used in this study. The membrane coupon was placed in the FO cell without a spacer, with the membrane’s active layer facing the feed side (AL–FS mode). Feed and draw solutions were circulated in corresponding closed loops on each side of the membrane in counter-current flow, and their flow rates were controlled by two peristaltic pumps (BT600–2J, LongerPump, Hebei, China) at ambient temperature (25 ± 2 °C). A magnetic stirrer (Benchmark Scientific Inc., Sayreville, NJ, USA) was used to homogenize the An-POME solution (to prevent sedimentation of suspended solids). The changes in DS weight were measured using an electronic weighing balance (GF-6100, A&D Company Limited, Tokyo, Japan), and the concentration changes of FS were measured using a conductivity meter (EC1100, Horiba Scientific, Kyoto, Japan). The concentrations of FS and DS were measured at the start and end of each experimental run. Both measuring instruments were connected to a computer where the measurements were monitored and logged at 5 min intervals (unless stated otherwise). The FO membrane system was circulated with DI water (both sides) for approximately 30 min to normalize the system’s temperature prior to the start of each experimental run. Then, the DI water was replaced with the FS and DS to be studied.

### 2.4. FO Performance Experiments

**Figure 2:**
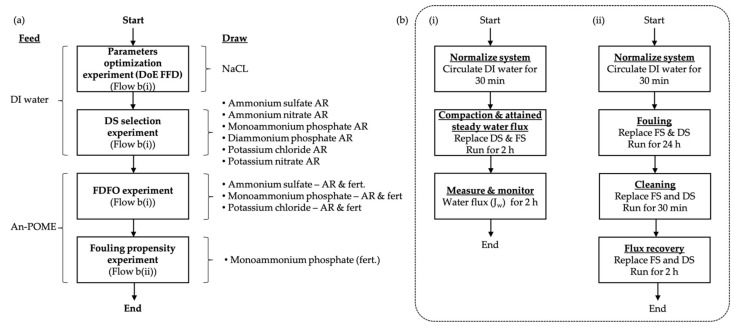
Process flow diagram for (**a**) Research experimental flow; (**b**) FO experiment where (**i**) Basic FO performance experiment; (**ii**) FO fouling propensity experiment.

The experimental flow of FO experiments is illustrated in Figure 2. Fresh membrane was used for each experimental run. Fresh FS and DS were used for each run according to experimental requirements. The water flux was measured continuously for all experiments, and the average water flux was determined after steady water flux was attained (i.e., after 2 h from the start of the run). All experiments lasted for 4 h, unless stated otherwise.

For the parameter optimization experiment, DI water and NaCl were used as the FS and DS, respectively. The FS volume of 1.0 L was fixed for all runs; the DS concentration, DS volume (hereafter refers as the FS to DS volume ratio (FS:DS)), and the system’s cross-flowrate were adjusted based on the parameter ranges of the factorial design of the experiment (DoE) as shown in Table 4. The baseline experiment was based on the optimized condition where the initial volumes of FS and DS were both 1.0 L, the DS concentration was 1 M, and the cross-flowrate was 600 mL/min (corresponds to a crossflow velocity of 11.09 cm/s).

The DS selection experiment utilized DI water as FS and reagent-grade chemical fertilizer DS to analyze the basic FO performance of the fertilizers. The FDFO experiments were carried out with An-POME as the FS and selected fertilizers (reagent and commercial-grade fertilizers) as DS to analyze the FDFO performance. The same conditions as the baseline experiment were applied to both experiments.

For the fouling propensity experiment, An-POME was used as the FS and MAP as DS. The baseline experimental conditions were applied for fouling and flux recovery runs. The system was left to operate for 24 h for fouling runs. After completion of fouling runs, two cleaning methods were applied separately to evaluate their effectiveness for water recovery. Hydraulic flushing, also known as membrane surface flushing, was conducted by replacing both FS and DS with DI water and recirculating for 30 min at a double cross-flowrate (i.e., 1200 mL/min) with reverse flow direction of the fouling run. Osmotic backwashing was employed where FS and DS were replaced with 1 M NaCl and DI water, respectively, to create a negative water flux. Osmotic backwashing was operated at a cross-flowrate of 600 mL/min for 30 min. After completing the cleaning run, the initial FS and DS were substituted back to the system for the flux recovery run for 2 h [16].

### 2.5. Measurement and Analysis

#### 2.5.1. Analytical Methods

The chemical oxygen demand (COD) and major elemental nutrient changes in the FS were monitored (i.e., total nitrogen (TN), total phosphorus (P) and potassium (K)) by determining the concentration using HACH tests and a spectrophotometer (DR3900, HACH, Ames, IA, USA). The total suspended solids (TSS) were determined by the gravimetric method. A Field Emission Scanning Electron Microscope, FE-SEM (Gemini SUPRA 55VP-ZEISS, Oberkochen, Germany) was used for analysing the membrane surface morphology. Fourier transform infrared spectroscopy, FTIR (Bruker, Billerica, MA, USA) was used for analysing the chemical properties of the membrane surface.

#### 2.5.2. Water Flux and Reverse Salt Flux

The basic FO performance was assessed in terms of water flux (J_W_) and reverse salt flux (J_S_). The water flux in the FO process is caused by the diffusion of water molecules due to an osmotic pressure difference across the membrane. At the same time, diffusion of the solute through an FO membrane also occurred, driven by a concentration difference between the feed and draw solutions [22]. The migration of water from the feed to the draw side during the FO process can be represented by the variation of the DS weight [23], J_W_ (L/m^2^ h), and is calculated using the equation:

(1) $$J_{w}=\frac{m_{D,t}-m_{D,0}}{\rho \;A\;\Delta t}$$

where m_D,t_ and m_D,0_ are the final and initial weight of DS (g) over a time period of Δt (h), ρ is the density of FS (g/L), and A is the effective membrane area (m^2^). Js (mmol/m^2^ h) can be determined by measuring the concentration changes of FS using the following equation [23]:

(2) $$\mathrm{Js}=\frac{\left(C_{D,t}V_{D,t}-C_{D,0}V_{D,0}\right)}{A\;\Delta t}$$

where Δt is the time interval, A is the effective membrane area, V_t_ and V_0_ are the final and initial volume of DS, respectively, and C_D,t_ and C_D,0_ are the concentration of the draw solute in the feed at final and initial times, respectively. When An-POME is used as feed, J_S_ was determined by analysing the reverse salt ion concentration in the FS at initial and final times in each experiment [16].

#### 2.5.3. Specific Reverse Salt Flux and Performance Ratio

The performance and suitability of the draw solution can be assessed in terms of the specific reverse salt flux (SRSF) and performance ratio (PR). SRSF is a ratio of RSF to the forward water flux (SRSF = J_S_/J_W_), and it is a good indicator for the estimation of salt losses from the DS during the FO process [9,14]. Having a DS with low SRSF is desirable for a sustainable FO operation as it relates to lower replenishment costs of the salts [24].

PR is a ratio of experimental water flux (J_W_) to the estimated theoretical water flux (J_Wt_) calculated as a percentage. It gives an indication of the bulk osmotic pressure accessible to effectively generate water flux across the FO membrane [14]. In the FO process, the osmotic pressure between the DS and FS is the main driver of water extraction; hence, the theoretical water flux was estimated using the following equation [25]:

(3) $$J_{\mathrm{Wt}}=A\left(\pi_{D,b}-\pi_{F,b}\right)$$

where A is the membrane’s pure water permeability coefficient, and π_D,b_ and π_F,b_ are the bulk osmotic pressures of the DS and FS, respectively. The osmotic pressures were estimated using OLI Stream Analyzer 9.6 software (OLI Systems, Inc., Paterson, NJ, USA).

#### 2.5.4. Water Recovery and Water Flux Recovery

Water recovery (Rec) is the percentage ratio of the water volume recovered from FS to the initial volume of FS (V_Rec_/V_F,0_), which gives an indication of the amount of water recovered from the FO process. The volume of water recovered (V_Rec_) was determined based on the mass of water that migrated from the FS to the DS and was calculated using the equation below [11]:

(4) $$V_{\mathrm{Rec}}={\;V}_{D,t}-V_{D,0}$$

(5) $$V_{\mathrm{Rec}}=\frac{m_{D,t}-m_{D,0}}{\rho }$$

where V_F,0_ is the initial volume of FS and other variables are as described previously.

The water flux recovery is the percentage ratio of the recovered flux after cleaning to the initial baseline flux (J_W,Rec_/J_W,b_). The flux was measured under the same conditions as the baseline experiment [6,11]. Flux recovery gave a measure of how efficient the membrane cleaning process was.

#### 2.5.5. Factorial Design of Experiment (DoE) for Parameter Optimization

The design of experiment (DoE) was applied to study and optimize the process parameters affecting FO performance. Parameter optimization was carried out by applying a two-level-three-factor (2^3^) full factorial design (FFD) of the experiment that was built and analyzed using Design-Expert version 12 software. The FO performance was assessed and optimized in terms of J_W_ and J_S_ that represent the responses. The low and high ranges and levels for the factors were selected based on literature [25,26,27], as shown in Table 4. The experiments were carried out in three replicates; hence, a total of 24 experiments, and in randomized order to avoid systematic errors, shown in Table S1. The regression analysis of the results and main and interaction effects between factors that affected the responses were determined by the software.

The influence of process parameters on the FO performance in terms of average water flux (J_W_) and reverse solute flux (J_S_) were evaluated statistically using Analysis of Variance (ANOVA) and factorial plots. The main factors and interactions of the experimental design were established using a linear model. The ANOVA and *p*-value significance levels were used to check the significance of the effect on both J_W_ and J_S_. Factorial plots, which consist of the main and interaction effect plots, normal probability plots, the surface plot, and the contour plot, showed how the parameters affected J_W_ and J_S_.

## 3. Results and Discussion

### 3.1. Effect of Process Parameters on FO Performance

#### 3.1.1. Analysis of Variance (ANOVA) and Normal Distribution

The experimental data of J_W_ and J_S_ are presented in Table S1. The ANOVA results for J_W_ and J_S_ are shown in Table 5 and Table 6, respectively. The model F-values for J_W_ and J_S_ were 162.8 and 16.07, respectively, implying that the models are statistically significant and there is only a 0.01% chance that this level of fit can occur due to noise. Based on the P-values at the level of significance of 0.05, A, B, C, and the two-way interaction AC had a statistically significant effect on J_W_. The R^2^ value of 0.9862 for J_W_ was acceptable, and the predicted R^2^ of 0.9688 is in reasonable agreement with the adjusted R^2^ of 0.9801. The same factors (A, C, and AC) were statistically significant for J_S_ except for B, which was insignificant with a *p*-value > 0.05. The R^2^ value of 0.8755 of J_S_ was still acceptable, and the Predicted R^2^ of 0.7199 is in reasonable agreement with the Adjusted R^2^ of 0.8210; i.e., the difference is less than 0.2.

The accuracy of the model can be further evaluated from the residuals of the factorial experiment, visualized by the normal probability plot of residuals. The data points for J_W_ were fairly close to the straight line, indicating that the experiments come from a normally distributed population. However, for J_S,_ the plot showed an “S” pattern along the line that indicated a slight deviation from the normal distribution, and the model might need transformation in order to make it more linear. The normal probability plots of residuals are presented in Figures S1 and S2.

#### 3.1.2. Main and Interactions Effects

**Figure 3:**
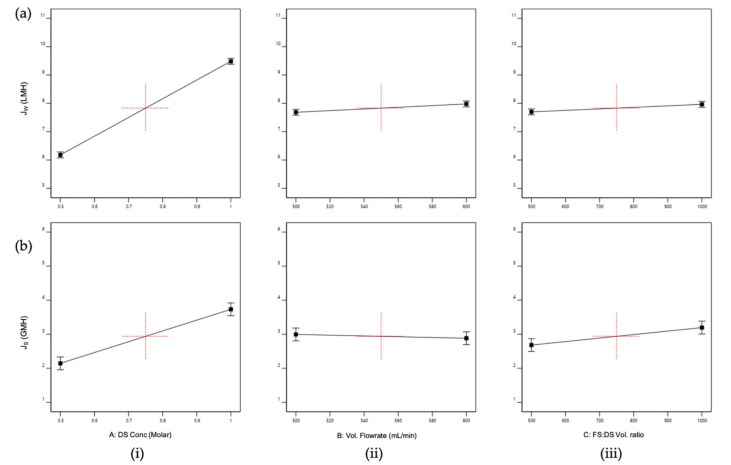
Main effects plots for (**a**) Average water flux, J_W_; (**b**) Reverse salt flux, J_S_; where the main effects are (**i**) Draw solution (DS) concentration, A; (**ii**) Volumetric flowrate, B; (**iii**) FS:DS volume ratio, C.

**Figure 4:**
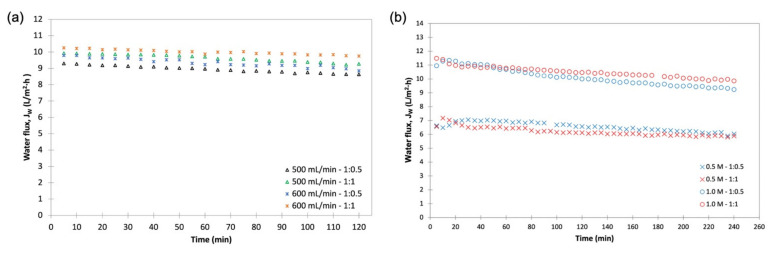
Pure water flux, J_W_, behaviors for (**a**) 1M NaCl, varying flowrate and FS:DS volume ratio (500 mL/min with a volume ratio of 1:0.5; 500 mL/min with a volume ratio of 1:1; 600 mL/min with a volume ratio of 1:0.5; 600 mL/min with a volume ratio of 1:1); (**b**) flowrate at 600 mL/min, varying NaCl concentration and FS:DS volume ratio (0.5 M NaCl with a volume ratio of 1:0.5; 0.5 M NaCl with a volume ratio of 1:1; 1.0 M NaCl with a volume ratio of 1:0.5; 1.0 M NaCl with a volume ratio of 1:1).

The sign of the main effects indicates the directions of the effect. All factors (i.e., DS concentration, volumetric flowrate, and FS:DS volume ratio) had positive effects on J_W_ (Figure 3a), where J_W_ increased as the factor changed from low to high levels. However, the DS concentration (Figure 3a(i)) had a greater degree of departure, indicating that the DS concentration was significant and affected J_W_, where J_W_ increased as the concentration increased. This is because in the FO process, the concentration difference (osmotic potential) is the driving force of the water migration from the feed to the draw side [28,29]. Although the flowrate is a significant factor for the model, it does not have an influence on J_W_ as shown by the almost horizontal effect plot (Figure 3a(ii)), supported by the minimal variation of J_W_ displayed in Figure 4a. This insignificant effect on J_W_ was expected, since concentrative external concentration polarization (ECP) was absent on the membrane’s active layer when DI water was used as FS [27]. The same effect was displayed by the FS:DS volume ratio towards J_W_ (Figure 3a(iii)) and can be seen in Figure 4b where at the same DS concentration, insignificant variation in water flux occurred at different volume ratios.

As for J_S_, the DS concentration and FS:DS volume ratio had a positive effect on J_S_ (Figure 3b(iii)), displaying the same trend seen for J_W_. The DS concentration had wide divergence from a low to a high level that indicates the DS concentration was significant, where J_S_ increased with increased concentration. This is true as the occurrence of reverse salt diffusion is due to the large solute concentration difference between the DS and FS [18]. The FS:DS volume ratio also had a slight divergence from a 1:0.5 to 1:1 volume ratio that signified it affected J_S_. However, the volumetric flowrate showed a negative effect (Figure 3b(ii)) and was the least significant towards J_S_, where it decreased with increased flowrate. At a higher flowrate, the boundary layer was thinner, which reduced the concentrated ECP and hence promoted a higher permeate flux [27]. This high permeate flux hindered the reverse diffusion of salt over to the feed side, resulting in lower J_S_.

**Figure 5:**
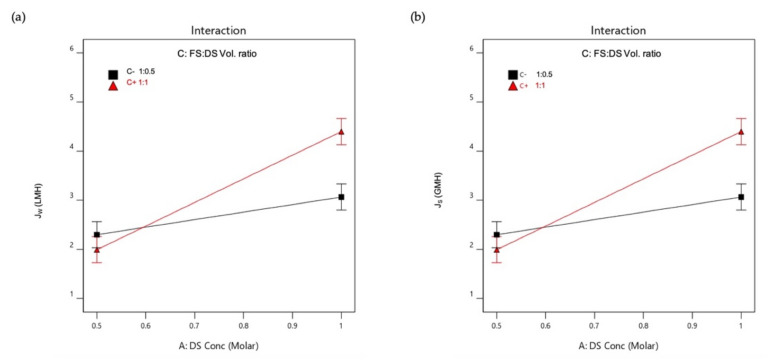
Interactions plot for (**a**) Average water flux, J_W_; (**b**) Reverse salt flux, J_S_.

**Figure 6:**
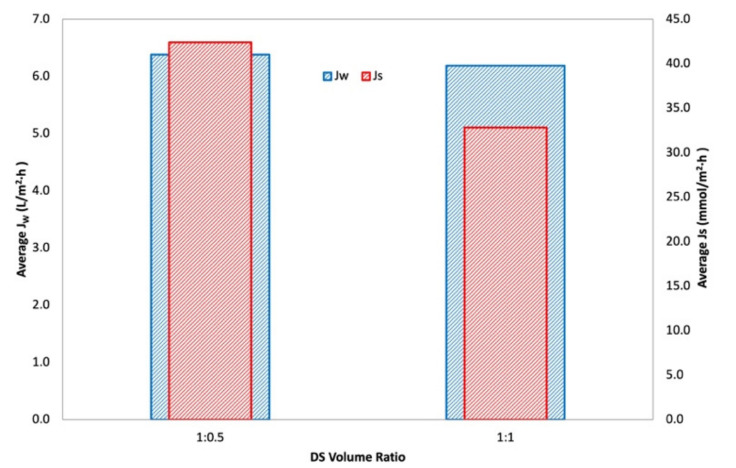
Average water flux, J_W_, and average reverse salt flux, Js, for 0.5 M NaCl, 600 mL/min, at a varying draw solution volume ratio (1:0.5 and 1:1).

**Figure 7:**
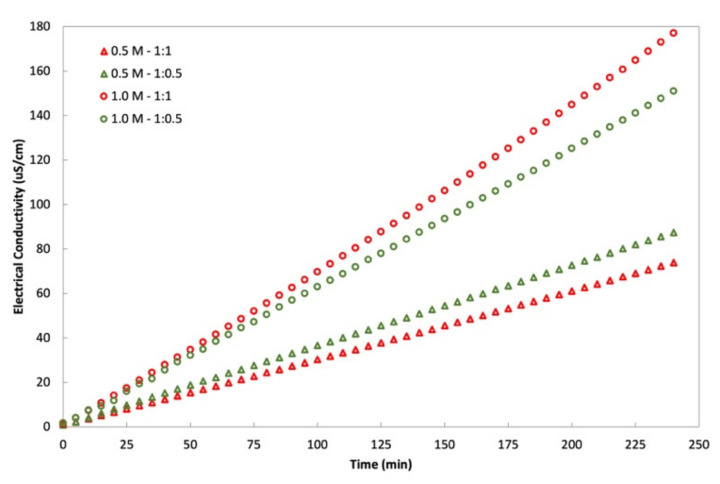
Feed solution (DI water) concentration with respect to time for varying NaCl concentrations and FS:DS volume ratio (0.5 M NaCl with a volume ratio of 1:0.5; 0.5 M NaCl with a volume ratio of 1:1; 1.0 M NaCl with a volume ratio of 1:0.5; 1.0 M NaCl with a volume ratio of 1:1).

An interaction is effective when the change in the response from low to high levels of a factor is dependent on the level of a second factor, that is when the lines do not run parallel. It can be seen from the plots in Figure 5 that the interaction between the DS concentration and FS:DS volume ratio was statistically significant in determining J_w_ and J_S_, given by the low and high levels crossing each other, where at a lower concentration, an FS:DS volume ratio of 1:0.5 gave slightly higher J_W_ and J_S_ than the 1:1 ratio. The slight variation in the water flux performance is attributed to concentration polarization (CP). In the FO process, water diffusion across the membrane from FS towards DS lowers the draw solute concentration inside the support layer more than the bulk solute concentration, hence giving rise to dilutive internal concentration polarization (ICP), and concentrative external concentration polarization (ECP) takes place on the feed-side membrane surface. In this case, a lower DS volume (1:0.5 ratio) has higher J_S_ compared to a higher DS volume (1:1 ratio) as shown in Figure 6. The high draw solute crossing over to the feed reduces the effect of dilutive internal concentration polarization inside the membrane, hence increasing the osmotic potential across the membrane. Consequently, a higher water flux was observed as portrayed in Figure 4b. Similar dilutive ICP phenomena also apply to J_S_. At a lower DS concentration, the 1:0.5 volume ratio gave higher RSF compared to 1:1. This finding was also supported by looking at the feed concentration trends with respect to time in Figure 7. Steeper gradients portrayed by the concentration curve for 0.5 M with the 1:0.5 DS volume ratio indicated the feed concentrated at a higher rate compared to the 1:1 volume ratio.

#### 3.1.3. Optimization

The parameter was optimized via numerical optimization using the constraints of maximum DS concentration, FS:DS volume ratio, J_W_, and J_S_, while the volumetric flowrate was set in the range. At the best point with maximum overall desirability of 0.7703, the optimum DS concentration, FS:DS volume ratio, and flowrate were 1.0 M, 1:1, and 600 mL/min, respectively. Under the optimum conditions, the water flux and RSF were 10.00 L/m^2^ h and 4.1677 g/m^2^ h, respectively. The cube plot of the optimum conditions generated is presented in Figures S1 and S2.

### 3.2. FO Performance of Draw Solutions—Initial Screening

Selection of an optimal DS is one of the crucial pieces in the FO puzzle. The two major criteria to consider in the selection of DS are DS with higher osmotic pressure than the FS to generate high water flux and having minimal reverse diffusion of salt from the DS to the FS. Other than these, the regeneration cost of DS is also essential for the FO process [18,28,29]. As for FDFO, regeneration cost does not come into play; instead, replenishment cost of the fertilizer used as DS will need to be considered. FO performance parameters analysed in terms of J_W_ and J_S_, performance ratio (PR), and specific reverse salt flux (SRSF) were used to assess and select the most suitable fertilizer as DS for the FDFO process.

**Figure 8:**
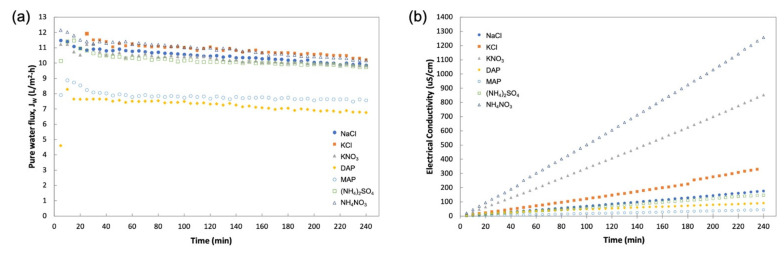
Profiles obtained during 4-h operation with DI water as FS and 1 M of various DSs for (**a**) pure water flux, J_W_; (**b**) FS concentration in electrical conductivity (EC) measurement.

**Figure 9:**
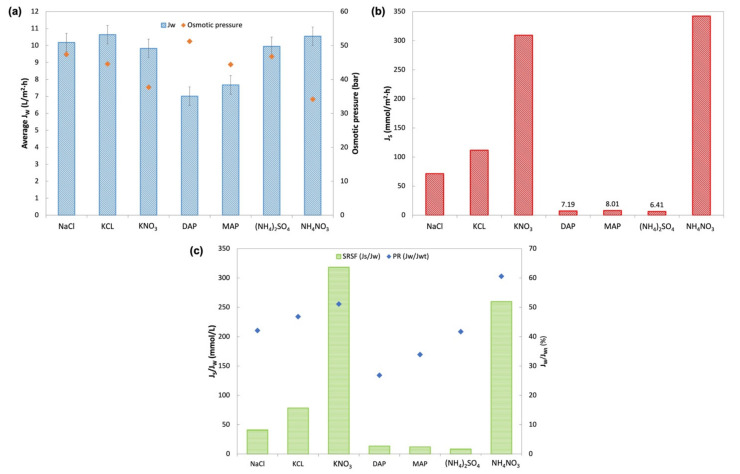
(**a**) Average water flux, J_W_, and osmotic pressure of the solution at 1 M concentration generated by OLI Stream Analyzer 9.6; (**b**) Reverse salt flux, J_S_; (**c**) Specific Reverse Salt flux, SRSF, and performance ratio (PR).

The DS selection experiment was carried out using reagent-grade fertilizers to evaluate their FO performance and also to compare with the baseline study carried out using NaCl as the DS. Figure 8 provides the profile for J_W_ and FS concentration for a 4-h operation. From Figure 8a, similar trends of pure water flux can be observed for all the DS fertilizers, with the only variations in water flux where MAP and DAP had lower J_W_ than the other solutions. Figure 8b displays a steady increase in the concentration of FS that indicated the reverse diffusion of salt from DS, with nitrate-based fertilizers (KNO_3_ and NH_4_NO_3_) having a steeper gradient compared to other fertilizers. These behaviors of water and solute flux were quantified by obtaining average J_W_ and J_S_ for better analysis, as shown in Figure 9a.

Pure water fluxes in the order from highest to lowest were KCl, NH_4_NO_3_, NaCl, (NH_4_)_2_SO_4_, KNO_3_, MAP, and DAP. KCl, KNO_3_, (NH_4_)_2_SO_4,_ and NH_4_NO_3_ all had J_W_ close to the baseline water flux of NaCl (10.18 L/m^2^ h). KCl DS showed the highest J_W_ of 10.64 L/m^2^ h, whereas MAP and DAP had the lowest water flux among all other fertilizers (7.68 L/m^2^ h and 7.01 L/m^2^ h, respectively). This is in agreement with findings from other studies on inorganic DS [14,18,24]. In theory, high osmotic pressure across the membrane would drive the FO process and produce high water flux. However, this is not reflected by MAP and DAP, which had low J_W_ despite having fairly high osmotic pressure (44.40 bar and 51.23 bar, respectively). The explanation behind this behavior relates to the concentration polarization effect, particularly the degree of ICP effects caused by the solute resistance (K) within the membrane support layer facing the DS [30]. Since K is strongly dependent on the diffusion coefficient (D) of the solute, a DS with higher D will have a lower K value and accordingly generate higher water flux [9,14,18,24]. Both MAP and DAP had the lowest diffusivity, accounting for the low water flux obtained. 

Figure 9c displays the performance ratio (PR) that suggests the availability of the bulk osmotic pressure for effective generation of water flux through the membrane. The trends of PR for the fertilizers slightly differ from the experimental J_W_ where NH_4_NO_3_ had the highest PR followed by KNO_3_, KCl, (NH_4_)_2_SO_4_, MAP, and DAP. All fertilizers showed a PR of more than 25%. The highest PR was for NH_4_NO_3_, with a PR of 61%, and the lowest was DAP with 27%. The trend in PR was found to be similar to previous studies by Phuntsho et al. [14,25].

The immense variation in RSF as portrayed in Figure 9b depends on the type of fertilizers used. DAP, MAP, and (NH_4_)_2_SO_4_ showed the lowest J_S_, while NH_4_NO_3_ had the highest J_S_ followed by KNO_3_. Generally, the ammonium compound of phosphate and sulfate gave better RSF than the chloride compound DS. On the other hand, this was not the case with NH_4_NO_3_, where both DSs with nitrate compounds displayed high RSF. Studies have shown that the RSF behavior observed is related to the ionic species of the DS and its hydrated ion radius (Table 7). Smaller hydrated ions more readily diffuse through the membrane compared to larger ions. Fertilizers with smaller hydrated ionic species of K^+^, NH^4+^, and Cl^−^, were found to have high RSF. Fertilizers with SO_4_^2−^ and PO_4_^2−^ that have a larger hydrated radius showed lower RSF regardless of their paired cations [18,24]. As for NO_3_^−^, despite having a larger hydrated radius, the high RSF indicated other factors such as inter-ionic effects might affect the movement of NO_3_^−^ across the membrane [31].

Specific reverse salt flux (SRSF) in Figure 9c shows that (NH_4_)_2_SO_4_ had the best SRSF of 8.5 mmol/L, followed by MAP and DAP with 12.0 L/mmol and 13.5 L/mmol of SRSF, respectively, while both nitrate compound fertilizers had the highest SRSF. SRSF demonstrates the amount of draw solute lost per unit volume of water extracted by the FO process [14]. Therefore, (NH_4_)_2_SO_4_ with an SRSF value of 8.5 mmol/L suggested that 8.5 mmol of (NH_4_)_2_SO_4_ was lost with every liter of water permeated from FS to the DS. In contrast, 318.2 mmol of KNO_3_ was lost with every liter of water extracted from FS. These findings showed that a small amount of (NH_4_)_2_SO_4_ was lost in the FO process compared to KNO_3_; hence, the replenishment cost for (NH_4_)_2_SO_4_ would be lower compared to that of other fertilizers. These findings are comparable to other studies on the potential of (NH_4_)_2_SO_4_, MAP, and DAP as DS based on their SRSF values [9,14,24].

From these observations, the optimum DS should have sufficiently good J_W_, low J_S,_ and low SRSF. (NH_4_)_2_SO_4_, MAP, and DAP were found to be good candidates as DS for further study. (NH_4_)_2_SO_4_ was chosen as the nitrogen source (N-source) fertilizer. MAP was selected over DAP as a phosphorus source fertilizer (P-source), as MAP showed better J_W_ and lower J_S_. KCl was the better potassium source (K-source) fertilizer compared to KNO_3_. Therefore, (NH_4_)_2_SO_4_, MAP, and KCl were further evaluated for FDFO experiments against An-POME as the feed.

### 3.3. FO Performance of Fertilizers as DS for the Treatment of Anaerobic POME

**Figure 10:**
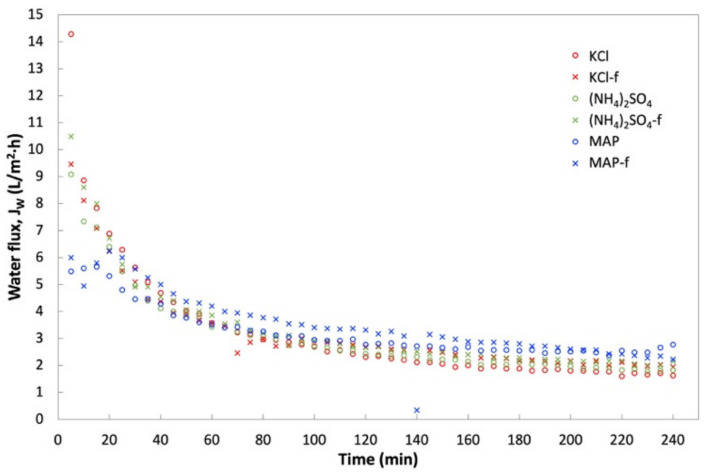
Water flux, J_W_, profile obtained during a 4-h operation with An-POME as FS and 1 M of various DS fertilizers.

**Figure 11:**
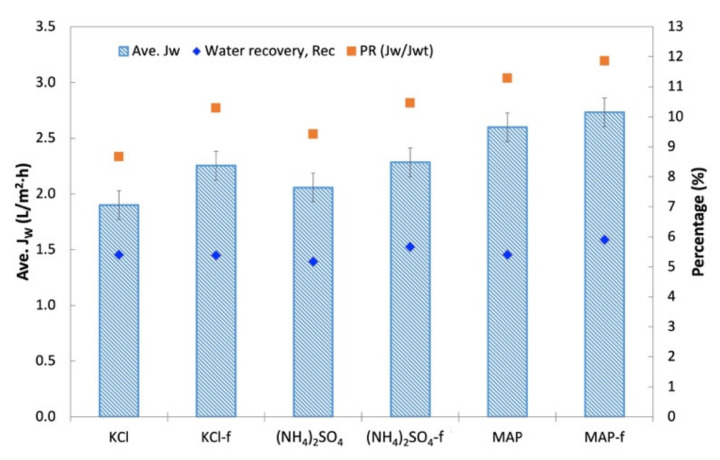
Average water flux, J_W_, of various DSs and corresponding water recovery, Rec, and percentage flux decline.

FDFO experiments were carried out using An-POME as feed against (NH_4_)_2_SO_4_, MAP, and KCl as the DS. Figure 10 shows the J_W_ behavior during the FDFO experiment for all the DSs evaluated. In general, J_W_ for all DSs showed a declining trend with a sharp drop during the first 70 min, then declined gradually and stabilized thereafter. The initial flux drop during the first 2 h of operation could be attributed to the deposition of impurities on the feed side of membrane surface, and salt built up in the FS due to RSF that increased the feed-side osmotic pressure and magnified fouling on the membrane surface due to a decline in the driving force, that in turn aggravated the CEOP effect [32,33]. The intensified CEOP effect gradually reduced the flux and eventually stabilized. Figure 11 shows the average J_W_ and its corresponding percentage flux decline (percent decrease of the initial J_W_ against the average J_W_). The average J_W_ for all the DSs ranged from 1.9 L/m^2^ h to 2.7 L/m^2^ h. Overall, commercial or technical-grade fertilizers have slightly higher average J_W_ compared to their respective reagent-grade counterparts. This variation might be related to the preparation of the DS that was based on molecular weight, which caused a slight variation of the initial concentration of the DS, as technical-grade chemicals contain some impurities. Both grades of MAP fertilizers had the highest average J_W_ and the lowest flux decline (below 54%), while all other DSs had fluxes that decreased by more than 70%. The higher flux decline seen for other fertilizers was most likely linked to the reverse salt diffusion towards FS [34], which was observed in an earlier study when DI water was used as the feed. 

In terms of water recovery, no significant variation was shown by all the DSs where the lowest and highest water recovery was 5.2% (for (NH_4_)_2_SO_4_) and 5.9% (for MAP-f). Although KCl and (NH_4_)_2_SO_4_ started off with a high initial J_W_ compared to MAP, MAP achieved a fairly high average J_W_ upon flux stabilization. This could impact the water extraction and compensate the fairly close water recovery value gained by all the DSs. In general, all technical-grade DSs (KCl-f, (NH_4_)_2_SO_4_-f and MAP-f) showed equal ability with their respective reagent-grade DS in terms of water recovery.

PR for An-POME as the feed was determined by using an osmotic pressure of 1.58 bar for An-POME, established by Johnson et al. [35], for theoretical water flux (J_Wt_) estimation. The PR trends for An-POME as feed were opposite those of the PR for DI water as feed. The PR of An-POME feed was significantly lower than the PR of DI water feed due to the high salt concentration in An-POME that lowered the osmotic potential between the feed and draw solution. Here, MAP had the highest PR followed by (NH_4_)_2_SO_4_ and KCl. Their respective commercial-grade solutions also displayed the same trends. The PR ranged from 9% (for KCl) to 12% (for MAP-f), which signifies sufficient bulk osmotic pressure for the effective drive of water diffusion through the membrane. Having a better PR contributed to MAP-f having a higher water recovery compared to the other DSs.

**Figure 12:**
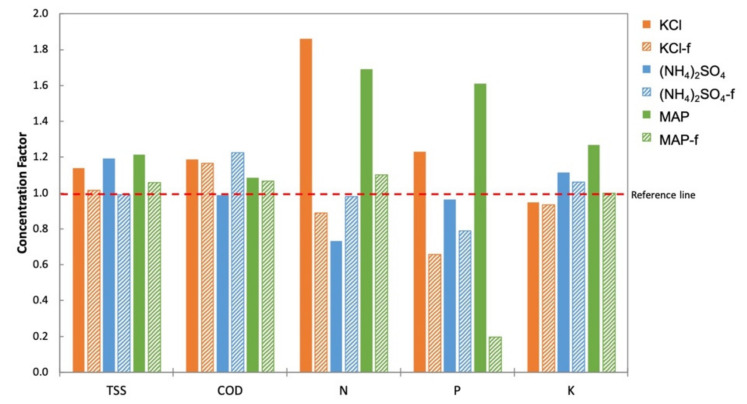
Concentration factor (final concentration/initial concentration) of total suspended solid (TSS), chemical oxygen demand (COD), and nutrients (nitrogen (N), phosphorus (P), and potassium (K)) in An-POME feed by various fertilizer DSs.

The TSS, COD, and nutrient (N, P, K) concentrations in the feed (An-POME) were measured prior to and after the FDFO experiment. The change in concentration of these parameters is presented in the form of a concentration factor (CF) displayed in Figure 12. The reference line in Figure 12 depicts CF = 1.0 that represents no concentration change. A CF value below the line indicates a reduction in concentration, and a CF value above the line means a concentration increase. The TSS concentration in feed is expected to increase during the water migration from FS to DS; the suspended solids were hindered by the high retention feature of the FO membrane. It can be seen that the TSS concentration increased for all DSs except (NH_4_)_2_SO_4_-f with a CF slightly below the reference line. Similar to TSS, COD also increased owing to the FO membrane characteristic of rejecting almost all types of organic matter. NaCl and (NH_4_)_2_SO_4_ showed a drop in concentration while the rest increased in COD. The disparity of nutrient concentrations in the feed was associated with the type of fertilizer DS and the feed water [24]. Nutrient concentrations exhibited variation, particularly for nitrogen and phosphorus that were possibly subjected to biodegradation of ammoniacal nitrogen (NH_4_^+^ -N) and phosphate-phosphorus (PO_4_^3−^ -P), respectively. Concentration changes in the salts in the feed (due to RSF from the DS and salt build-up by the concentrating effect of FS) affected the saline-sensitive microbes in An-POME that drives biomass activity [36]. A more pronounced concentration drop observed for N and P of (NH_4_)_2_SO_4_ with the lowest RSF, indicated lower accumulation of NH_4_^+^ and SO_4_^2−^ that subsequently reduced the osmotic stress in the feed and improved the biomass activity for NH_4_^+^ -N degradation.

### 3.4. Fouling Behavior and Water Flux Recovery

**Figure 13:**
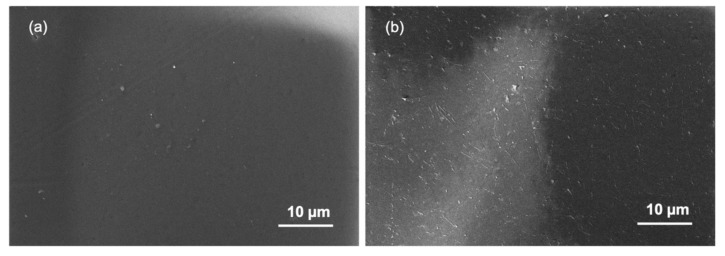
Field Emission Scanning Electron Microscopy (FESEM) images with 1000× magnification on the active layer of the cellulose triacetate (CTA) membrane: (**a**) pristine membrane and (**b**) fouled membrane.

**Figure 14:**
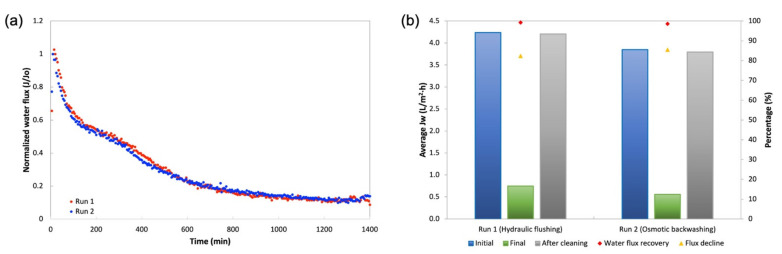
(**a**) Normalized water flux (J/J_0_) decline profile obtained during a 24-h operation for two runs under same conditions with An-POME as FS and 1 M MAP as DS; (**b**) Average water flux in the initial stage (first 2 h of operation), final stage (final 2 h of operation), after cleaning, flux decline, and water flux recovery after hydraulic flushing and osmotic backwashing.

The studies of membrane fouling were conducted using An-POME as feed and a 1M MAP solution as the DS and later were subjected to two physical cleaning methods individually: hydraulic flushing and osmotic backwashing. The initial (for both fouling and flux recovery after physical cleaning) and final average J_W_ were determined from the first 2 h of operation and final 2 h of operation, respectively. Figure 13 shows the FESEM images of the active layer surface of a pristine membrane (Figure 13a) and fouled membrane (Figure 13b). It can be seen that the pristine CTA membrane had a smooth surface. After fouling with An-POME, the surface was fully covered with organic foulant. Table 8 summarizes the results for the fouling and cleaning experimental runs. Figure 14a displays the normalized water flux (J/J_0_) behavior during the 24-h operation for two experimental runs under the same operating conditions. A steep declining gradient can be seen up to about 2 h, after which the flux dropped moderately for about 10 h and then started to stabilize towards the end of the operation. Similar behavior was previously explained in Section 3.3. The slight variation in flux behavior between runs 1 and 2 was due to a temperature difference and fluctuation during the experiments that were conducted at ambient temperature (temperature = 25 ± 2 °C). From Figure 14b, the flux decline for long-term fouling was around 84%, which was shown by the large drop of average J_W_ from the initial flux of 4.24 L/m^2^ h and 3.85 L/m^2^ h to a final flux of 0.75 L/m^2^ h and 0.56 L/m^2^ h, for run 1 and run 2, respectively. The water recovered from run 1 and run 2 was 16.4% and 12.7%, respectively, for a 24-h operation. 

Two physical cleaning methods were employed to remove foulants on the membrane surface. Hydraulic flushing for 30 min with double the crossflow velocity (CFV) of the baseline experiment managed to achieve 99.2% water flux recovery. A study by Ansari et al. [37] achieved full water flux recovery by hydraulic flushing at a CFV of five times the baseline experiment and was found to be the most effective strategy compared to other cleaning methods (air-scouring and ultrasonic application). It is implied that shearing was induced on the fouling layer by the high CFV; its effectiveness was attributed to the dislodging of foulant from the membrane surface and the absent of water permeation during cleaning as both sides of the membrane were replaced with DI water. 

Osmotic backwashing was able to recover 98.6% of the initial water flux, which was slightly lower than that by hydraulic flushing. However, previous studies showed that for organic foulants, osmotic backwashing performs better than hydraulic flushing, as it is able to remove foulants within the support layer [36,37,38]. The changes in water flow direction by switching the DS with lower osmotic pressure (DI water) than the FS (1M NaCl) also allows the effective dislodging of foulants from the membrane surface [39]. The discrepancy of the findings from this study compared to other studies could be related to the severity of fouling that depends on the period of long-term fouling conducted, the characteristics of the feed, and the type of membrane used. Nevertheless, both hydraulic flushing and osmotic backwashing were able to effectively restore the water flux but did not achieve complete flux recovery.

**Figure 15:**
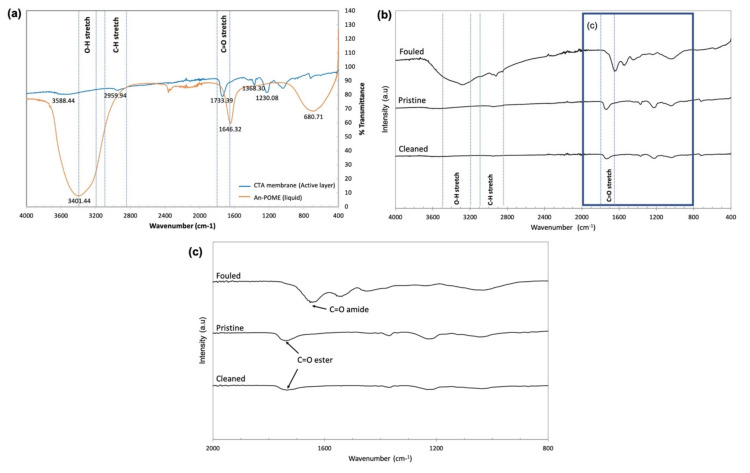
Fourier transform infrared spectroscopy (FTIR): (**a**) spectra of a pristine CTA membrane (active layer) and liquid An-POME; (**b**) stacked spectra of pristine, fouled, and cleaned CTA membranes on the active layer; (**c**) zoomed in at wavenumber 800–2000 cm^−1^ for stacked spectra of pristine, fouled, and cleaned CTA membranes on the active layer.

The fouling phenomena and cleaning efficiency can be understood by looking at the changes in the membrane surface functional groups. Figure 15 displays the FTIR spectra of liquid An-POME and the membrane used in the fouling experiments. Figure 15a shows the main functional groups of liquid An-POME and the active layer of a pristine CTA membrane for use as reference to analyze the efficiency of the cleaning. For liquid POME, the broad rounded and very strong peak of the band located between 3000 and 3700 cm^−1^ indicates variations in O–H bonds that might originate from the hydroxyl group and water (the moisture content after anaerobic digestion was higher than 90%) or amine group [40,41]. The broad peak suggests the presence of carboxylic acid where the carboxylic functional group arises from long chain fatty acid (LCFA), as palmitic acid (C_16_H_32_O_2_) and oleic acid (C_18_H_34_O_2_) are the major fatty acids found in POME [42]. The band between 2850 and 3100 cm^−1^ shows the presence of C–H bonds where a peak higher than 3000 cm^−1^ indicated the alkene C=H, whereas peaks less than 3000 cm^−1^ indicated alkane C–H [43], where both could be found in the LCFA. A sharp strong peak detected at 1646 cm^−1^ was attributed to the presence of an amide carbonyl group (C=O amide). The broad band around 681 cm^−1^ could denote aromatic C–H bending. 

For the active layer of the CTA membrane, the major spectral feature was the band around 1733 cm^−1^ that corresponds to the ester carbonyl group (C=O ester), and other characteristic peaks around 1042 and 1230 cm^−1^ can be assigned to asymmetric and symmetric stretching of C–O bonds [43,44]. The small shoulder around 2960 cm^−1^ accounted for the C–H bonds [45]. Figure 15b compares the AL surface of the CTA membrane for pristine, fouled, and cleaned membranes. The fouled membrane surface depicts the presence of a major functional group band found in An-POME: hydroxyl group (O–H around 3293 cm^−1^), C–H bond (around 2922 cm^−1^), and amide carbonyl group (C=O at around 1652 cm^−1^). Figure 15c further differentiates between the carbonyl group where the peaks usually resided around 1630 and 1690 cm^−1^ for C=O amide, whereas C=O esters were detected around 1735 and 1750 cm^−1^. It can be seen that there was no large difference between the spectra for pristine and cleaned membranes, except for the lower absorption intensity of the cleaned membrane, which reflects the inability to obtain complete water flux recovery after physical cleaning has been applied. 

A study by Choi et al. [46] on a hybrid FO–RO (reverse osmosis) seawater desalination process found that the flux and recovery of FO membranes are crucial factors for commercialization of the FO process. In a pilot-scale study by El Zayat et al. [47], it was discovered that by applying FDFO as a post-treatment of brine desalination using RO, the CAPEX (Capital expenditure) of the RO plant decreased by USD 20.29 per m^3^ of the plant’s capacity. The OPEX (Operational expenditure) had a reduction of the brine disposal cost: USD 0.08 for every cubic meter of the final diluted DS. With respect to POME treatment, a study with biogas capturing using a covered anaerobic lagoon was estimated to have a CAPEX of $5,649,000, OPEX $3,217,000, and a net present value (NPV) of $2,830,000 [48]. An FO-based open-loop wastewater reclamation revealed that an MBR–FO–RO gave a ~20% NPV benefit over the classical MBR–RO. Based on these findings, it can be assumed that the application of FO or FDFO for An-POME could have an economic advantage by reducing the NPV of the processing plant. Furthermore, taking into account the value added from resource recovery and sustainable operation of the plant would make it more economically attractive.

## 4. Conclusions

This study investigated the potential of the FDFO process using commercial fertilizers to treat An-POME for nutrient and water recovery. Factors influencing the FO process, suitable fertilizers as DS, FO performance, and fouling propensity were investigated. The performance of six fertilizers as DSs was assessed in terms of J_W_ and J_S_, the performance ratio (PR), and the specific reverse salt flux (SRSF). The major outcomes from this study are:

- With DI water feed, KCl and NH_4_NO_3_ showed highest average J_W_; however, (NH_4_)_2_SO_4_, MAP, and DAP demonstrated the lowest J_S_ and SRSF. The optimum DSs with sufficiently good average J_W_, low J_S,_ and low SRSF were (NH_4_)_2_SO_4_, MAP, and DAP.
- With An-POME feed, (NH_4_)_2_SO_4_, MAP, and KCl were chosen as the N-source, P-source, and K-source fertilizer, respectively, for DS. Although it started out with a lower initial J_W_ than KCl, both reagent and technical grades of MAP showed high average J_W_ and low flux decline due to the ability to sustain a relatively high water flux throughout the process. MAP also had higher PR compared to other DSs that indicated a sufficient osmotic gradient for driving water removal from An-POME.
- Generally, no significant changes in TSS and COD in the An-POME feed were observed, as the FDFO process only involves water migration. Significant variation was observed in nutrient (NPK) concentrations in the feed that are associated with the type of fertilizer DS (salinity build up due to RSF from the draw solution) and the characteristics of the initial feed.
- The long-term fouling using An-POME feed and MAP as DSs yielded a flux decline of around 84% and an average 15% water recovery for a 24-h operation. Sufficient cleaning using hydraulic flushing and osmotic backwashing was able to effectively restore the water flux, although complete flux recovery was not achieved, with 99.2% and 98.6% water flux recovery, respectively.

Further study is still required to investigate how the nutrient concentration changes (due to RSF), especially NPK in An-POME and fertilizer DS, after the FDFO process would affect agriculture application. Further dilution of DS is expected for direct use in fertigation, as reported by other similar FDFO studies [25,31]. Long-term low fouling operation also needs to be addressed for a success FDFO application. Nevertheless, FDFO using commercial fertilizers for water reuse and nutrient recovery from An-POME has a promising future application.

## Figures and Tables

**Table 1:** The cellulose triacetate (CTA) membrane characteristics.

Water Permeability Coefficient, A (LMH/bar)	Salt Permeability Coefficient, B (LMH)	Structural Parameter, S μm
0.51	0.156	480

**Table 2:** Characteristics of anaerobically treated palm oil mill effluent (An-POME).

Parameters	Concentration (mg/L)
pH	7.76
Chemical oxygen demand (COD)	13.646
Total suspended solid (TSS)	7.701
Total Nitrogen (TN)	617
Phosphorus (P)	1.264
Potassium (K)	5.450

**Table 3:** Details and properties of fertilizers used as draw solutions.

Chemicals	Chemical Formula	Molecular Weight, MW (g/mol)	Supplier	Osmotic Pressure (bar) ^a^	Diffusivity, D (10^−9^ m^2^/s) ^a^
Reagent grade:					
Sodium chloride	NaCl	58.44	Chemiz, Malaysia	47.39	
Ammonium sulfate	(NH_4_)_2_SO_4_	132.14	Systerm	46.75	1.14
Ammonium nitrate	NH_4_NO_3_	80.04	Hamburg Chemical	34.13	1.65
Monoammonium phosphate	NH_4_H_2_PO_4_	115.03	Chemiz, Malaysia	44.40	1.06
Diammonium phosphate	(NH_4_)_2_HPO_4_	132.06	Chemiz, Malaysia	51.23	0.912
Potassium chloride	KCl	74.56	Systerm	44.55	1.79
Potassium nitrate	KNO_3_	101.10	Chemiz, Malaysia	37.68	1.78
Commercial grade:					
Ammonium sulfate	(NH_4_)_2_SO_4_	132.14 ^b^	n/a	-	-
Monoammonium phosphate	NH_4_H_2_PO_4_	115.03 ^b^	n/a	-	-
Muriate of potash (MOP)	KCl	74.56 ^b^	Behn-Meyer Agricare	-	-

^a^ The thermodynamic properties at 1 M concentration and 25 °C were generated by using OLI Stream Analyzer 9.6. ^b^ MSDS was not provided by supplier; thus, the MW of this commercial grade chemical was assumed to be the same as its respective reagent grade.

**Table 4:** Experimental ranges and levels of the factors used in the full factorial design (FFD).

Factors	Coded Symbol	Range and Level	
		Low (−)	High (+)
DS concentration (Molar)	A	0.5	1.0
Flowrate (mL/min)	B	500	600
FS:DS volume ratio	C	1:0.5	1:1

**Table 5:** ANOVA for average water flux: effect of DS concentration (A), DS volumetric flowrate (B), and FS:DS volume ratio (C).

Source	Sum of Squares	df	Mean Square	F-Value	*p*-Value	
Model	67.40	7	9.63	162.80	<0.0001	significant
A-DS Conc.	65.42	1	65.42	1106.01	<0.0001	
B-Vol. Flowrate	0.5207	1	0.5207	8.80	0.0091	
C-FS:DS Vol. ratio	0.4248	1	0.4248	7.18	0.0164	
AB	0.0452	1	0.0452	0.7634	0.3952	
AC	0.9886	1	0.9886	16.71	0.0009	
BC	0.0071	1	0.0071	0.1202	0.7334	
ABC	0.0018	1	0.0018	0.0308	0.8630	
Pure Error	0.9463	16	0.0591			
Cor Total	68.35	23				

R^2^ = 0.9862, Adjusted R^2^ = 0.9801, Predicted R^2^ = 0.9688.

**Table 6:** ANOVA for J_S_: effect of DS concentration (A), DS volumetric flowrate (B), and FS:DS volume ratio (C).

Source	Sum of Squares	df	Mean Square	F-Value	*p*-Value	
Model	21.26	7	3.04	16.07	<0.0001	significant
A-DS Conc.	15.09	1	15.09	79.90	<0.0001	
B-Vol. Flowrate	0.0745	1	0.0745	0.3943	0.5389	
C-FS:DS Vol. ratio	1.58	1	1.58	8.38	0.0106	
AB	0.0556	1	0.0556	0.2942	0.5950	
AC	4.04	1	4.04	21.38	0.0003	
BC	0.4121	1	0.4121	2.18	0.1591	
ABC	0.0004	1	0.0004	0.0021	0.9642	
Pure Error	3.02	16	0.1889			
Cor Total	24.28	23				

R^2^ = 0.8755, adjusted R^2^ = 0.8210, predicted R^2^ = 0.7199.

**Table 7:** Hydrated radius of ionic species involved in this study, adapted from Ref. [31].

Ion	Hydrated Radius (nm)
Cation:	
K^+^	0.201
Na^+^	0.178
NH^4+^	0.104
Anion:	
Cl^−^	0.195
NO^3−^	0.340
SO_4_^2−^	0.300
PO_4_^2−^	0.339

**Table 8:** Results for fouling and cleaning experimental runs.

Run	Average Water Flux, J_W_ (L/m^2^ h)			Water Flux Recovery (%)	Flux Decline (%)	Water Recovery (%)
	Initial	Final	After Cleaning			
Run 1 (Hydraulic flushing)	4.24	0.75	4.20	99.2	82.4	16.4
Run 2 (Osmotic backwashing)	3.85	0.56	3.80	98.6	85.5	12.7

## Data Availability

Not applicable.

## Supplementary Materials

The following are available online at https://www.mdpi.com/article/10.3390/membranes11080566/s1, Figure S1: Normal probability plot of residuals for (a) Average water flux, JW; (b) Reverse salt flux, JS, Figure S2: FCube plot of optimum conditions for (a) Desirability (b) Average water flux, JW; (c) Reverse salt flux, JS, Table S1. Experimental design matrix and results of 2^3^ FFD.
