# Supplementary material for: Evaluating Fertilizer-Drawn Forward Osmosis Performance in Treating Anaerobic Palm Oil Mill Effluent
Source: Membranes (Basel). 2021 Jul 28;11(8):566. doi: 10.3390/membranes11080566 (PMC8401652; doi:10.3390/membranes11080566)
Supplement: Supplementary file 1 [file membranes-11-00566-s001.zip › membranes-1298848-supplementary.pdf]

membranes

Supplementary Material

Evaluation of Fertilizer-drawn Forward Osmosis for the Treatment of Anaerobic Palm Oil Mill Effluent Using Commercial Fertilizer as Draw Solution

Ruwaida Abdul Wahid [1], Wei Lun Ang [1,2]*, Abdul Wahab Mohammad [1,2], Daniel James Johnson [3] and Nidal Hilal [3]

[1] Department of Chemical and Process Engineering, Faculty of Engineering and Built Environment, Universiti Kebangsaan Malaysia, 43600 Bangi, Selangor Darul Ehsan, Malaysia
[2] Centre for Sustainable Process Technology (CESPRO), Faculty of Engineering and Built Environment, Universiti Kebangsaan Malaysia, 43600 Bangi, Selangor Darul Ehsan, Malaysia
[3] NYUAD Water Research Center, New York University Abu Dhabi, P.O. Box 129188, Abu Dhabi, United Arab Emirates
* Correspondence: wl_ang@ukm.edu.my

Appendix A

Table S1. Experimental design matrix and results of 2^3 FFD.

Run	Level			J_W	J_S
	A	B	C	LMH	GMH
1	-	+	+	6.126	1.825
2	+	-	-	9.132	3.372
3	-	+	+	6.177	1.898
4	+	+	-	9.607	3.515
5	+	-	-	8.714	2.933
6	+	-	-	8.995	2.819
7	-	+	-	6.594	2.734
8	-	-	+	5.965	2.05
9	+	-	+	9.999	5.55
10	+	+	-	8.964	2.406
11	+	+	-	9.465	3.342
12	+	-	+	9.29	3.628
13	+	+	+	9.607	4.162
14	+	+	+	10.218	4.246
15	+	-	+	9.619	4.71
16	-	-	-	6.296	2.389
17	-	+	-	6.279	2.387
18	-	-	-	6.054	2.138
19	+	+	+	10.176	4.095
20	-	-	-	6.011	1.987
21	-	-	+	6.138	2.118
22	-	+	+	6.262	1.825
23	-	+	-	6.267	2.158
24	-	-	+	5.994	2.236

Appendix B

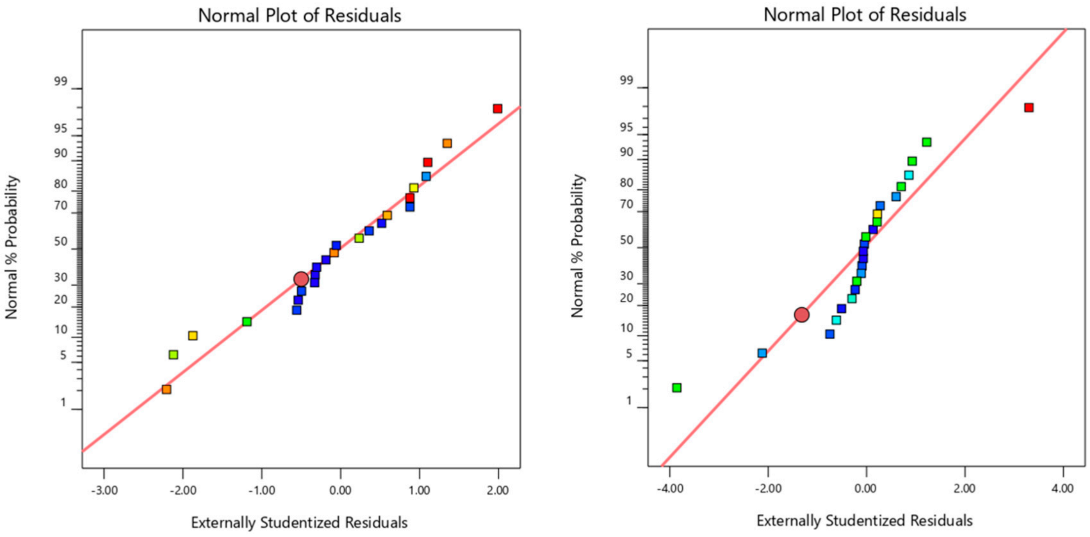

Figure S1. Normal probability plot of residuals for: (a) Average water flux, J_W; (b) Reverse salt flux, J_S.

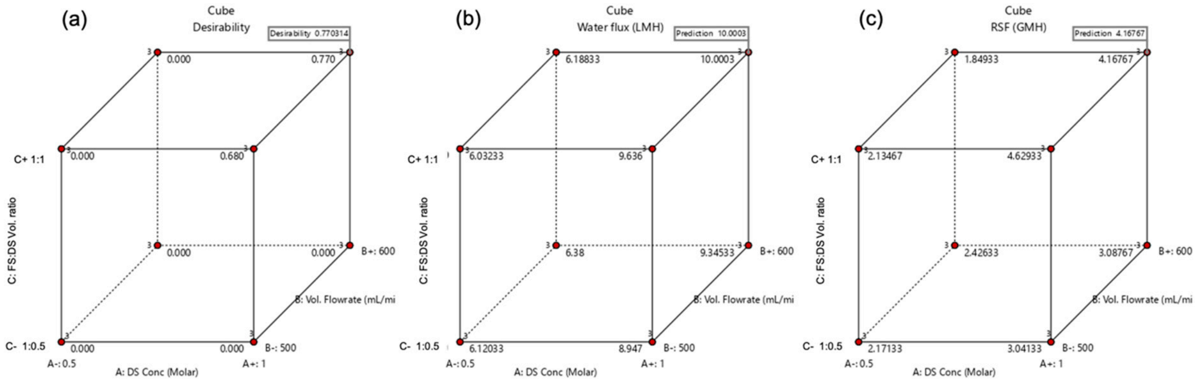

Figure S2. Cube plot of optimum conditions for: (a) Desirability (b) Average water flux, J_W; (c) Reverse salt flux, J_S.
